# Health-care leaders’ and professionals’ experiences and perceptions of compassionate leadership: A mixed-methods systematic review

**DOI:** 10.1108/LHS-06-2023-0043

**Published:** 2023-10-16

**Authors:** Kevin Östergård, Suvi Kuha, Outi Kanste

**Affiliations:** Research Unit of Health Sciences and Technology, University of Oulu, Oulu, Finland; Research Unit of Health Sciences and Technology, University of Oulu, Oulu, Finland and Finnish Centre for Evidence-Based Health Care, Helsinki, Finland

**Keywords:** Compassionate, Compassionate leadership, Health care, Leaders, Leadership, Systematic review

## Abstract

**Purpose:**

The purpose of this study is to identify and synthesise the best evidence on health-care leaders’ and professionals’ experiences and perceptions of compassionate leadership.

**Design/methodology/approach:**

A mixed-methods systematic review was conducted in accordance with the Joanna Briggs Institute methodology for mixed-methods systematic reviews using a convergent integrated approach. A systematic search was done in January 2023 in PubMed, CINAHL, Scopus, Medic and MedNar databases. The results were reported based on Preferred Reporting Items for Systematic Reviews and Meta-analyses. The data was analysed using thematic analysis.

**Findings:**

Ten studies were included in the review (five qualitative and five quantitative). The thematic analysis identified seven analytical themes as follows: treating professionals as individuals with an empathetic and understanding approach; building a culture for open and safe communication; being there for professionals; giving all-encompassing support; showing the way as a leader and as a strong professional; building circumstances for efficient work and better well-being; and growing into a compassionate leader.

**Practical implications:**

Compassionate leadership can possibly address human resource-related challenges, such as health-care professionals’ burnout, turnover and the lack of patient safety. It should be taken into consideration by health-care leaders, their education and health-care organisations when developing their effectiveness.

**Originality/value:**

This review synthesised the knowledge of compassionate leadership in health care and its benefits by providing seven core elements of health-care leaders’ and professionals’ experiences and perceptions of compassionate leadership.

## Introduction

Health-care services worldwide are currently facing several complex changes and challenges, such as a skilled labour shortage (Anderson *et al.*, 2021) and professionals’ burnout and turnover (Kelly *et al.*, 2021). The global COVID-19 pandemic has also challenged health-care services and leadership (Raso *et al.*, 2021). Leadership trends have increasingly emphasised the importance of modern, soft-skilled (Leclerc *et al.*, 2020; Abraham *et al.*, 2021) and human-centred leadership, which has been proposed as a substitute for typical top-down linear leadership in health care (Leclerc *et al.*, 2020). The importance of high-quality, safe and compassionate care has been recognised in health-care organisations, and compassionate leadership has been introduced as the catalyst to meet these needs (de Zulueta, 2015).

Compassion has been described as a crucial attribute of health-care leadership (de Zulueta, 2015). In health care, compassion itself has been defined as a deep awareness and understanding of another’s suffering, concerns, pain and distress. Compassion is distinguished from empathy by motivation and the need to help and take action to relieve one’s suffering (Strauss *et al.*, 2016), while empathy usually only refers to the understanding of others’ perspectives, reactions and feelings (Niedtfeld, 2017).

Compassionate leadership can be defined as a dynamic process of shared and distributed leadership involving altruism and the need to help others. However, similar behaviour and abilities are present also in other leadership styles and especially in authentic, transformational, resonant, servant and adaptive leadership but unlike other leadership styles, compassionate leadership emphasises compassion (de Zulueta, 2015). Compassionate leadership can also be conceptualised through different contents. De Zulueta (2021) identified four key elements of compassionate leadership – attending, empathising, understanding and helping – while Shuck *et al.* (2019) identified the essence of a compassionate leader through six themes – integrity, accountability, presence, empathy, authenticity and dignity. To build and promote compassionate leadership in health-care organisations, it is important that leaders practice compassionate leadership (de Zulueta, 2015). Compassionate leadership can be learnt through experience, personal growth, professional development and education (Carragher and Gormley, 2017).

Compassionate leadership can affect employee well-being and reduce burnout (Lown *et al.*, 2020) by alleviating negative emotions, such as anxiety, burnout and workplace behavioural deviances (Choi *et al.*, 2016). Compassionate leadership may also help ease the suffering of health-care workers in stressful and difficult times, such as during the COVID-19 pandemic (Oruh *et al.*, 2021; Sánchez-Romero *et al.*, 2022).

Leaders’ compassion-giving can increase employees’ self-esteem, self-efficacy (Choi *et al.*, 2016), job satisfaction and performance (Chen *et al.*, 2022). Receiving compassion is also negatively associated with an intention to leave (Chen *et al.*, 2022; Choi *et al.*, 2016). Compassion can be very demanding, and compassion-related stress or compassion fatigue can be a barrier to providing compassion (Papadopoulos *et al.*, 2020). Other obstacles to compassion-giving are related to leaders’ values, personality, organisational culture (Singh *et al.*, 2018) and cultural or geographical background (Papadopoulos *et al.*, 2020). Leaders may fear losing authority and professionalism if they use a compassionate approach. Stress and burnout are also system-related obstacles to compassion giving, and they are often related to the high demands of managerial work (Papadopoulos *et al.*, 2020).

On an organisational level, compassion is enabled and supported by leaders who value and expect compassion. Organisational compassion is related to improving organisational, professional and patient outcomes (Lown *et al.*, 2020). The need for organisational compassion has increased, as it leads to better experiences and a more thriving workforce. Consequently, organisational cultures should be developed towards a culture of compassion, where health-care leaders play an important role (de Zulueta, 2021; Vogus and McClelland, 2020). Health-care leaders can promote and support compassion in organisations by role modelling for compassion and by having leadership for compassion (Straughair, 2019). Compassion leads to high-quality relationships with employees, and it helps employees to be more compassionate towards patients (Vogus and McClelland, 2020).

A preliminary search from PROSPERO, the Cochrane Database of Systematic Reviews and *JBI Evidence Synthesis* was conducted, and no current or in-progress systematic reviews of compassionate leadership in health care were identified. In a systematic review, Ramachandran *et al.* (2023) conceptualised compassionate leadership at a general level and identified six critical dimensions of compassionate leadership – empathy, openness and communication, physical, mental health and well-being, inclusiveness, integrity and respect and dignity. A scoping review by Evans (2022) explored current knowledge about compassionate leadership in health care. The review highlighted that compassionate leadership connected to patient care outcomes and could play a significant role in addressing change in health care. Based on these findings, it is important for health-care organisations to strive towards compassionate leadership, and it is important to identify how health-care leaders’ and professionals’ experience and perceive compassionate leadership.

Thus, the purpose of this review was to identify and synthesise health-care leaders’ and professionals’ experiences and perceptions of compassionate leadership. The review question was as follows: What kinds of experiences and perceptions do health-care leaders and professionals have about compassionate leadership?

## Research methods

### Study design

A mixed-methods systematic review (MMSR) was conducted in accordance with the Joanna Briggs Institute (JBI) methodology for MMSRs using a convergent integrated approach (Lizarondo *et al.*, 2020). The review was registered in PROSPERO, the International Prospective Register of Systematic Reviews (CRD42022383919). The results were described based on the Preferred Reporting Items for Systematic Reviews and Meta-analyses (PRISMA) (Page *et al.*, 2021) (Supplementary Table 1).

### Data sources and search strategy

A three-step search strategy was used to find both published and unpublished studies. Firstly, an initial limited search of PubMed was undertaken, followed by an analysis of the text words contained in the title and abstract and the index terms of relevant articles. Secondly, a full literature search was conducted by three researchers (authors blinded) in four electronic databases (PubMeb, CINAHL, Scopus and the Finnish database Medic) in January 2023 (Table 1). Sources of unpublished studies and grey literature were searched from MedNar. The search strategy was developed in cooperation with an information specialist. Finally, the reference list of the included studies was screened for additional studies.

### Search outcomes

**Figure 1.**
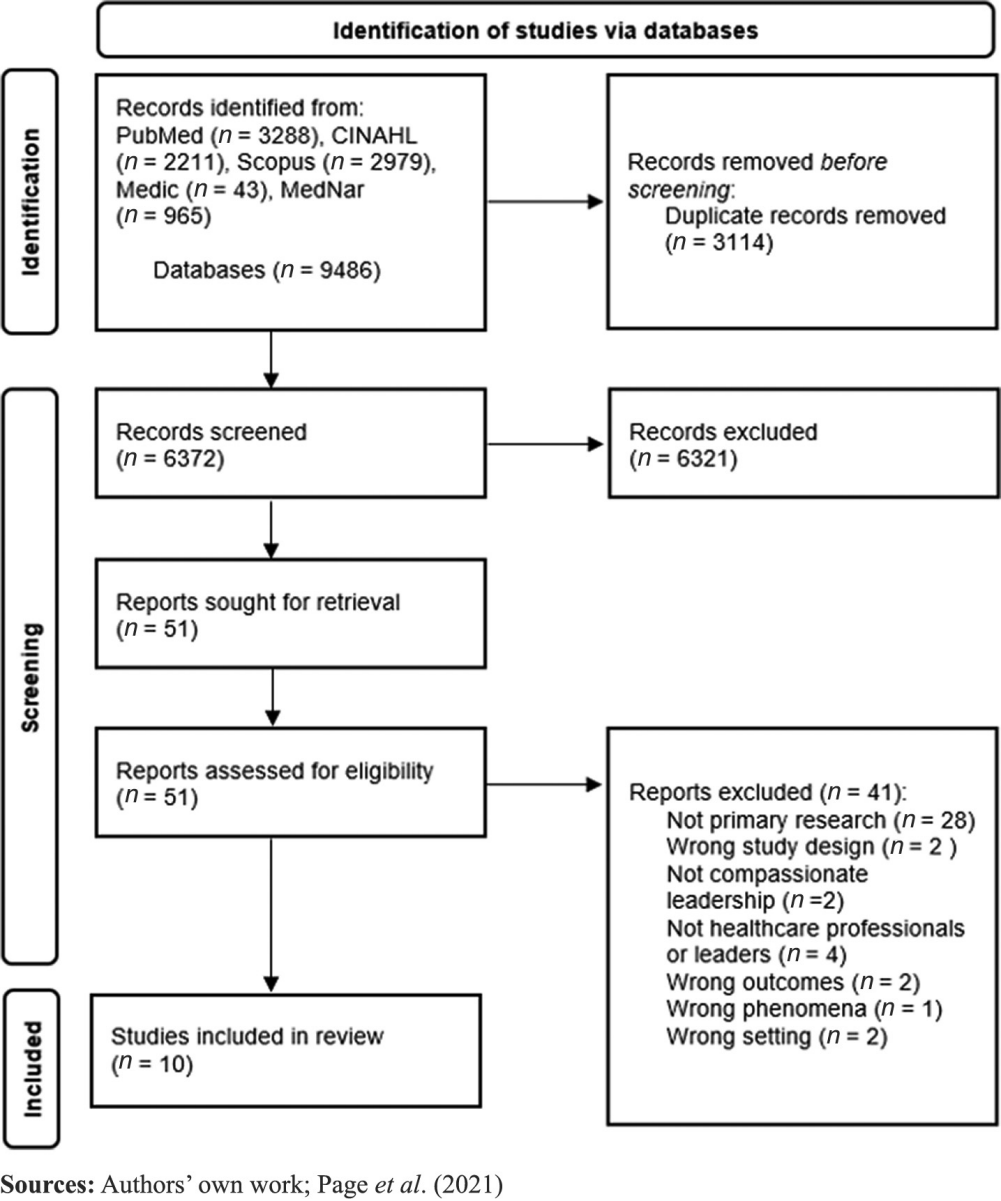
Illustration of the search process and study selection in PRISMA flow diagram

The inclusion and exclusion criteria were defined using PICo (Participants, Phenomena of interest and Context) (Table 2). All identified citations were uploaded into the bibliographic management system Covidence (version 2.0). The database search identified 9,486 studies, and after removing duplicates, a total of 6,373 articles were screened for titles and abstracts by three independent reviewers. Any disagreements that arose between the reviewers were resolved through discussion or with a third reviewer. A total of 6,321 articles were excluded as irrelevant, and the full texts of 51 articles were retrieved by two independent reviewers (KÖ, SK). Of these, 10 articles met the inclusion criteria and were included for critical appraisal. No further studies were identified through manual searches. A list of the excluded full-text studies is provided in Supplementary Table 2. Of the included studies, five were qualitative and five were quantitative. The results of the search are presented in the PRISMA flow diagram (Figure 1).

### Quality assessment

Eligible studies were critically appraised for methodological quality according to the JBI guidelines by two independent reviewers (KÖ, SK). Qualitative studies were evaluated according to the Critical Appraisal Checklist for Qualitative Research (Lockwood *et al.*, 2020), and quantitative studies were evaluated according to the critical appraisal checklist for analytical cross-sectional studies (Moola *et al.*, 2020). Any disagreements that arose between the reviewers were resolved through discussion or with a third reviewer (OK).

All the studies were included in the systematic review after a critical appraisal of the methodological quality. The included qualitative studies scored 7–9/10, and the included quantitative studies scored 6–8/8 (Supplementary Tables 3 and 4).

### Data extraction and synthesis

Quantitative and qualitative data were extracted from the included studies by one reviewer (KÖ) using Microsoft Word software (Microsoft Corporation, Redmond, WA). The data extraction was crosschecked with other reviewers. The extracted data included the author, year of publication, county of origin, study purpose, participants, methods of data collection and analysis and key findings (Supplementary Table 5).

The quantitative data was converted into qualitative data following the JBI convergent integrated approach for MMSR by extracting the quantitative data and translating it into textual data based on the research questions by repeated detailed examination (Lizarondo *et al.*, 2020). Data was collected from two of the quantitative studies (Salminen-Tuomaala and Seppälä, 2022a; Sansó *et al.*, 2022), and three quantitative studies (López-Díaz *et al.*, 2022; Papadopoulos *et al.*, 2021; Papadopoulos *et al.*, 2022) had already reported their findings in a qualitative form. The qualitative data were then pooled with the data from the qualitative studies, allowing quantitative and qualitative data to be combined (Stern *et al.*, 2021) (Supplementary Table 6). All the experiences and perceptions were pooled together, regardless of the participants of the studies.

Three reviewers were involved in the data synthesis using thematic analysis (Thomas and Harden, 2008). The manual synthesis followed a three-step process that combined qualitative and qualitative data. Firstly, the extracted initial codes were coded line by line (*n* = 233). Secondly, the grouped codes were organised into descriptive themes (*n* = 55) by similarities. In the last stage, descriptive themes were developed into sub-themes (*n* = 18) and analytical themes (*n* = 7).

## Findings

### Characteristics of the studies

The included studies were conducted in the UK (*n* = 3), Finland (*n* = 2), Australia (*n* = 1), Spain (*n* = 1) and Colombia (*n* = 1), and two (*n* = 2) were conducted in several countries. The participants were health-care leaders (*n* = 1,315) and health-care professionals (*n* = 368). The number of participants ranged from 8 to 50 in qualitative studies and from 50 to 1,217 in quantitative studies.

### Synthesis of the findings

The thematic analysis identified seven analytical themes that combined the experiences and perceptions of health-care leaders and professionals on compassionate leadership – treating professionals as individuals with an empathetic and understanding approach; building a culture for open and safe communication; being there for professionals; giving all-encompassing support; showing the way as a leader and as a strong professional; building circumstances for efficient work and better well-being; and growing into a compassionate leader (Table 3).

### Treating professionals as individuals with an empathetic and understanding approach

The analytical theme of treating professionals as individuals with an understanding and an empathetic approach comprised three sub-themes – leaders considering individuality, empathy and understanding.

Professionals experienced and perceived individuality as a need to be treated as a human being (Ali and Terry, 2017; Hewison *et al.*, 2019; López-Díaz *et al.*, 2022; Salminen-Tuomaala and Seppälä, 2022b) and that their private needs were met (Hewison *et al.*, 2019; López-Díaz *et al.*, 2022; Salminen-Tuomaala and Seppälä, 2022b; Sansó *et al.*, 2022). The individual approach also included a focus on the personalities of individuals and knowing them (Hewison *et al.*, 2019; O'Toole *et al.*, 2021; Papadopoulos *et al.*, 2022; Salminen-Tuomaala and Seppälä, 2022b), which included observations such as awareness of employees’ circumstances (Hewison *et al.*, 2019; Papadopoulos *et al.*, 2022) and person-centred focus (O'Toole *et al.*, 2021).

Showing empathy (Hewison *et al.*, 2019; Papadopoulos *et al.*, 2022; Salminen-Tuomaala and Seppälä, 2022a; Salminen-Tuomaala and Seppälä, 2022b; Sansó *et al.*, 2022) and having emotional intelligence (Ali and Terry, 2017; Hewison *et al.*, 2018; Hewison *et al.*, 2019; O'Toole *et al.*, 2021; Papadopoulos *et al.*, 2022; Sansó *et al.*, 2022) are crucial factors in compassionate leadership. It was also expected that leaders would be compassionate towards professionals (Ali and Terry, 2017; López-Díaz *et al.*, 2022; O'Toole *et al.*, 2021; Salminen-Tuomaala and Seppälä, 2022a; Salminen-Tuomaala and Seppälä, 2022b; Sansó *et al.*, 2022). Perceptions of not receiving empathy or being allowed to show negative feelings were also mentioned (Salminen-Tuomaala and Seppälä, 2022a).

The leader’s understanding was emphasised and experienced and perceived as a general feeling that professionals were understood (Hewison *et al.*, 2019; Papadopoulos *et al.*, 2021; Papadopoulos *et al.*, 2022; Salminen-Tuomaala and Seppälä, 2022b; Sansó *et al.*, 2022). Moreover, leaders’ understanding appeared to be an awareness of the work and its demands (Hewison *et al.*, 2019; Salminen-Tuomaala and Seppälä, 2022b). The burden of understanding (Hewison *et al.*, 2019; Salminen-Tuomaala and Seppälä, 2022b) can be experienced as exhausting (Salminen-Tuomaala and Seppälä, 2022b). Understanding itself can be missing or inadequate (Salminen-Tuomaala and Seppälä, 2022a; Salminen-Tuomaala and Seppälä, 2022b) based on the experiences and perceptions of health-care leaders and professionals.

### Building a culture for open and safe communication

The analytical theme of building a culture for open and safe communication comprises two sub-themes – listening skills and open and authentic discussion.

The meaning of listening was emphasised in compassionate leadership, and listening skills included the feeling that professionals were being heard (Hewison *et al.*, 2018; López-Díaz *et al.*, 2022; Papadopoulos *et al.*, 2021; Papadopoulos *et al.*, 2022; Salminen-Tuomaala and Seppälä, 2022b; Sansó *et al.*, 2022) and that listening was mutual (Salminen-Tuomaala and Seppälä, 2022b). Professionals who had a feeling of being heard perceived that the leader was a sensitive and active listener (Papadopoulos *et al.*, 2021; Papadopoulos *et al.*, 2022) who knew how to listen (López-Díaz *et al.*, 2022) and took professionals’ concerns seriously (Papadopoulos *et al.*, 2022). Mutual listening was experienced as listening to each other (Salminen-Tuomaala and Seppälä, 2022b).

According to the results, a compassionate leader encourages open and authentic discussion. This was experienced and perceived through sincere conversations (Ali and Terry, 2017; Papadopoulos *et al.*, 2021; Papadopoulos *et al.*, 2022; Salminen-Tuomaala and Seppälä, 2022b), such as honest and courageous discussions (Ali and Terry, 2017) and genuine dialogue (Salminen-Tuomaala and Seppälä, 2022b), and by promoting communication and information sharing (Papadopoulos *et al.*, 2022; Salminen-Tuomaala and Seppälä, 2022b). Difficulties in communication were also reported, and they were experienced as a lack of dialogue and the feeling of not being heard (Salminen-Tuomaala and Seppälä, 2022b).

### Being there for professionals

The analytical theme of being there for professionals comprised two sub-themes – presence and participation and being approachable and open.

The presence and participation of the leader were expected from compassionate leadership. The leaders’ presence and participation in the work (Papadopoulos *et al.*, 2021; Papadopoulos *et al.*, 2022; Salminen-Tuomaala and Seppälä, 2022b; Sansó *et al.*, 2022) was perceived as attending (Papadopoulos *et al.*, 2022; Sansó *et al.*, 2022), as in the physical presence of the leaders and participation in daily routines (Salminen-Tuomaala and Seppälä, 2022b). Knowing what is going on in the work unit (Sansó *et al.*, 2022; Salminen-Tuomaala and Seppälä, 2022b) and working in cooperation with professionals (Papadopoulos *et al.*, 2022; Sansó *et al.*, 2022) were also perceived as important parts of the leaders’ presence and participation.

Being approachable and open was also mentioned as an important trait of a compassionate leader. Leaders being easy to approach (Papadopoulos *et al.*, 2021; Papadopoulos *et al.*, 2022; Salminen-Tuomaala and Seppälä, 2022b) and being open to different views (Papadopoulos *et al.*, 2022; Salminen-Tuomaala and Seppälä, 2022b) were considered important. Personal virtues of the leader (Ali and Terry, 2017; Hewison *et al.*, 2018; Papadopoulos *et al.*, 2022; Salminen-Tuomaala and Seppälä, 2022b; Sansó *et al.*, 2022), such as being pleasant and kind (Salminen-Tuomaala and Seppälä, 2022b), warm (Sansó *et al.*, 2022) and fair (Papadopoulos *et al.*, 2022) approach, were reported as important to the leader. Approachability can also be experienced as difficult if the leader is distant and silent or has deficient manners (Salminen-Tuomaala and Seppälä, 2022b).

### Giving all-encompassing support

The analytical theme of giving all-encompassing support comprised three sub-themes – personalised support, support in professional growth and making professionals feel meaningful.

The results emphasised the significance of support in compassionate leadership. Personalised support was experienced and perceived as supporting the balance between work and personal life (Hewison *et al.*, 2019; Papadopoulos *et al.*, 2022; Salminen-Tuomaala and Seppälä, 2022b) and giving emotional support to professionals (Hewison *et al.*, 2018; Hewison *et al.*, 2019; Salminen-Tuomaala and Seppälä, 2022b). Being helpful in different ways, such as solving problems to help others (Sansó *et al.*, 2022), was also emphasised. Leaders also perceived that when they gave support to professionals, they in turn received support back from professionals (Papadopoulos *et al.*, 2021).

Leader’s support of professional growth was experienced and perceived as supporting professional development (Hewison *et al.*, 2019; Papadopoulos *et al.*, 2021; Papadopoulos *et al.*, 2022; Salminen-Tuomaala and Seppälä, 2022b), building the confidence and resilience of professionals (Hewison *et al.*, 2018; Hewison *et al.*, 2019; Salminen-Tuomaala and Seppälä, 2022b), challenging professionals in a positive way (Hewison *et al.*, 2019; Salminen-Tuomaala and Seppälä, 2022b) and giving positive and constructive feedback (Papadopoulos *et al.*, 2022; Salminen-Tuomaala and Seppälä, 2022b). The professionals’ descriptions of the leaders’ support varied. Professionals mentioned that they received either a lack of or inadequate support from leaders (Salminen-Tuomaala and Seppälä, 2022a; Salminen-Tuomaala and Seppälä, 2022b).

The results show that professionals experienced the need to feel meaningful, and it was important that the leader made them feel that way. Making professionals feel meaningful included showing appreciation (Hewison *et al.*, 2018; Papadopoulos *et al.*, 2021; Salminen-Tuomaala and Seppälä, 2022b) and giving recognition (Hewison *et al.*, 2018; López-Díaz *et al.*, 2022). Meaningfulness was also reported when leaders made professionals feel valued (Hewison *et al.*, 2018; Papadopoulos *et al.*, 2021; Papadopoulos *et al.*, 2022), empowered (Hewison *et al.*, 2018; Hewison *et al.*, 2019; Papadopoulos *et al.*, 2021; Papadopoulos *et al.*, 2022) and when leaders were being encouraging to professionals (Papadopoulos *et al.*, 2022; Salminen-Tuomaala and Seppälä, 2022a; Salminen-Tuomaala and Seppälä, 2022b).

### Showing the way as a leader and as a strong professional

The analytical theme of showing the way as a leader and as a strong professional comprised three sub-themes – being a mentor and a good example, being open and handling difficult situations transparently and being advocative and a fair professional.

The sub-theme of being a mentor and a good example consisted of acting as a role model and leading by example (Ali and Terry, 2017; Hewison *et al.*, 2018; Papadopoulos *et al.*, 2021). The results show that it is important for leaders to demonstrate compassionate care (Ali and Terry, 2017; Hewison *et al.*, 2019). Creating a culture of care (Hewison *et al.*, 2019) and having the courage to challenge behaviours that are not compassionate towards patients (Ali and Terry, 2017) were experienced as some of the ways for leaders to demonstrate compassionate care. Impulsive and inconsistent leading (Salminen-Tuomaala and Seppälä, 2022b) were mentioned as obstacles to role modelling and leading by example.

The meaning of open and transparent handling of difficult situations was emphasised. Solving problems and resolving conflicts (O'Toole *et al.*, 2021; Papadopoulos *et al.*, 2021; Papadopoulos *et al.*, 2022; Salminen-Tuomaala and Seppälä, 2022b; Sansó *et al.*, 2022) were experienced and perceived, for example, as open problem management (Salminen-Tuomaala and Seppälä, 2022b) and as trying to understand problems (Sansó *et al.*, 2022). The results included a sensitive approach to mistakes (Papadopoulos *et al.*, 2022; Salminen-Tuomaala and Seppälä, 2022b; Sansó *et al.*, 2022), which was perceived as a sensitive and tactful handling of mistakes and avoiding blame culture (Papadopoulos *et al.*, 2022).

The results indicate that being an advocative and fair professional also represents a compassionate leader. Standing up for professionals was perceived when a leader acts as a staff advocate (López-Díaz *et al.*, 2022; Papadopoulos *et al.*, 2021; Papadopoulos *et al.*, 2022) and defends employees (López-Díaz *et al.*, 2022). Acting as a professional (Ali and Terry, 2017; Hewison *et al.*, 2019; Salminen-Tuomaala and Seppälä, 2022b) included experiences of consistency in the leader’s actions like not having to be afraid of the leader’s mood affecting his or her behaviour (Ali and Terry, 2017).

### Building circumstances for efficient work and better well-being

The analytical theme of building circumstances for efficient work and better well-being comprised three sub-themes – knowing the team and using the team in an adequate way; enhancing the operational environment; and relation to overall well-being.

Knowledge of the team and use of the team in an adequate way consisted of actively promoting teamwork (Ali and Terry, 2017; Papadopoulos *et al.*, 2021; Papadopoulos *et al.*, 2022; Salminen-Tuomaala and Seppälä, 2022b) and recognition and using team members’ strengths (Salminen-Tuomaala and Seppälä, 2022a). In some cases, it was also perceived that the leader did not recognise employee or team strengths, including perceptions such as the leader not appreciating everybody’s competence (Salminen-Tuomaala and Seppälä, 2022a).

The experiences and perceptions show that compassionate leaders enhanced the operational environment by building an environment of respect (López-Díaz *et al.*, 2022; Papadopoulos *et al.*, 2022; Salminen-Tuomaala and Seppälä, 2022b) and by building a united work community (Hewison *et al.*, 2018; López-Díaz *et al.*, 2022; Papadopoulos *et al.*, 2021; Salminen-Tuomaala and Seppälä, 2022b). According to the results, compassionate leadership is related to the quality of care and patient safety (Hewison *et al.*, 2018; López-Díaz *et al.*, 2022; Papadopoulos *et al.*, 2021; Salminen-Tuomaala and Seppälä, 2022b).

It was also experienced that compassionate leadership is related to overall well-being by better contentment and decreasing the risk of burnout. These were experienced as enhanced well-being, better psychological safety at the workplace and reduced stress (Salminen-Tuomaala and Seppälä, 2022b). The risk of burnout was perceived to be reduced for both leaders and professionals, and compassionate leadership was described as beneficial for mental health, enhanced professional life quality and satisfaction for both leaders and professionals. Compassionate leadership was also perceived to be related with manager–staff relationships, as these relationships became closer, more positive and open (Papadopoulos *et al.*, 2021).

### Growing into a compassionate leader

The analytical theme of growing into a compassionate leader comprised two sub-themes – building compassionate leadership skills and having self-help abilities.

The perceptions show that compassionate leadership skills can be built by having experience in leadership. Furthermore, compassionate leadership can also be learnt through leadership education and personal development (Salminen-Tuomaala and Seppälä, 2022a). The results indicate that having self-help abilities has an impact on growing into a compassionate leader. Having self-help abilities included knowing and taking care of oneself and the ability to be self-compassionate. Self-help abilities were experienced and perceived as taking action for psychical and psychological self-care (Sansó *et al.*, 2022) and self-awareness (Ali and Terry, 2017; Sansó *et al.*, 2022). The ability to self-compassionate was emphasised as the ability to experience the concept of compassion themselves (O'Toole *et al.*, 2021) and perceived as having positive self-compassion (Sansó *et al.*, 2022).

## Discussion

This review introduced seven original and unique core elements of health-care leaders’ and professionals’ experiences and perceptions of compassionate leadership. These core elements have not been identified in previous studies, although some parts of these elements have been previously recognised.

Our results showed that compassionate leadership is a broad and diverse concept that health-care leaders and professionals can describe in different ways. Some of the findings were contradictory, indicating that many situational factors can influence leadership behaviour. The findings suggest that treating professionals as individuals with an empathetic and understanding approach constitutes a core element of compassionate leadership. Individuality, empathy and understanding should all be constantly visible in the behaviour of the leader, but these needs increase especially during stressful times, such as the COVID-19 pandemic (Salminen-Tuomaala and Seppälä, 2022a). Similar findings concerning stressful times have also been made in other studies (Oruh *et al.*, 2021; Sánchez-Romero *et al.*, 2022). In addition, Moudatsou *et al.* (2020) stated that empathy among health-care professionals significantly contributes to the behaviour and overall well-being of professionals. The findings of our review highlight the need and longing for compassion among health-care professionals. Papadopoulos *et al.* (2020) noted that compassion can be demanding and that compassion-related stress or compassion fatigue can be an obstacle to giving compassion. Leaders should take this into consideration and recognise their own limits before compassion becomes a burden.

Our findings revealed that building a culture of open and safe communication requires leaders who are compassionate and who hear their employees out. The feeling of being heard was considered crucial to employee well-being (Hewison *et al.*, 2018; López-Díaz *et al.*, 2022; Papadopoulos *et al.*, 2021; Papadopoulos *et al.*, 2022; Salminen-Tuomaala and Seppälä, 2022b; Sansó *et al.*, 2022), and this requires that the leader is a sensitive, active (Papadopoulos *et al.*, 2021; Papadopoulos *et al.*, 2022) and an attentive listener (Sansó *et al.*, 2022). Moreover, our review shows that listening skills and encouraging open discussion lead to a culture of open and safe communication. In a previous study by de Zulueta (2021), the importance of meaningful communication as a part of compassionate leadership was also highlighted.

The findings of our review highlight the significance of a leader being there for professionals as a core element of compassionate leadership. Professionals wanted their leaders to be present and to be involved in the work. Based on our findings, a compassionate leader was described as approachable and open. Leader’s presence (Shuck *et al.*, 2019) and attendance (de Zulueta, 2021) have also been described as key elements of compassionate leadership in some previous studies.

According to our core elements, giving all-encompassing support is emphasised in compassionate leadership. According to de Zulueta (2021), support can also be defined as helping, which is a key element of compassionate leadership. This review shows that professionals expect personalised support in a broad range of matters, such as when facing adversaries in their private lives (Salminen-Tuomaala and Seppälä, 2022b), in distress (Hewison *et al.*, 2018) and in emotional work (Hewison *et al.*, 2019). Our results showed that making professionals feel meaningful was an important part of support from compassionate leadership as well as supporting professional development. De Zulueta (2021) also noted that it is important for professionals to feel that they have a purpose and meaning in their work.

Role modelling and showing the way as a leader and as a strong professional were experienced as important according to the core elements. A compassionate leader should be an example that every professional wants to follow. These findings are similar to Straughair’s (2019) results, which argue that role modelling is significant for leaders and that it should have a compassion-focused approach. Leaders should demonstrate compassion and create a compassionate care culture.

This review indicates that a compassionate leader can build circumstances for efficient work and better well-being, which efficiency comes from knowing the team and everybody’s competence. This can also enhance the operational environment and result in a better quality of care and patient safety. Lown *et al.* (2020) also noted that compassion has a positive effect on patient outcomes. Our findings showed that compassionate leadership is related to better well-being, and these findings are similar to Lown *et al.*’s (2020) results, which address that compassionate leadership is linked to employee well-being and to reducing burnout. Moreover, in previous studies, compassion has been found to improve well-being by increasing job satisfaction and performance (Chen *et al.*, 2022) and to ease employee anxiety and burnout (Choi *et al.*, 2016).

The results of this review suggest that it is possible to grow into a compassionate leader. This can be achieved through personal development, experience and education (Salminen-Tuomaala and Seppälä, 2022a). These findings are aligned with the findings of Carragher and Gormley (2017), who identified the possibility of achieving compassionate leadership through personal growth. Our review suggests that self-help abilities are linked to being a compassionate leader, and educational programmes could benefit from focusing on these skills.

Unlike previous descriptions of compassionate leadership in health care, this review highlighted that a compassionate leader could build a culture for open and safe communication, better circumstances for efficient work and that a compassionate leader shows the way as a leader and as a strong professional. Ramachandran *et al.* (2023) have similar findings of communication and well-being but on a general level and not from a health-care context. Other previously found dimensions, such as integrity, accountability, authenticity, dignity (Shuck *et al.*, 2019), inclusiveness, integrity and respect (Ramachandran *et al.*, 2023), were not identified as core elements in this review.

This review showed that the need for compassion in leadership is obvious and that compassion is not always present in leaders’ actions (Salminen-Tuomaala and Seppälä, 2022a; Salminen-Tuomaala and Seppälä, 2022b). Sánchez-Romero *et al.* (2022) also found that health care needs programmes for leaders that promote healthy and compassion-based behaviours because professionals’ suffering can be managed with a compassionate approach. The need for compassion-focused leadership education is also noted by Straughair (2019), who suggested that nurse leaders should have skills, knowledge and attributes to foster a compassionate culture. Empathetic skills should be educated through continuous and personal development programmes (Moudatsou *et al.*, 2020).

### Limitations

This MMSR was based on a protocol made in forehand that was followed carefully. Three researchers were involved in every phase of the review process, and a library information specialist was consulted when developing the search strategy. There was a chance of bias with the search strategy and language limitations. The quality of the included studies was evaluated by using JBI critical appraisals, and all studies scored well above half. Data extraction and analysis were conducted by one researcher, increasing the possibility of subjectivity in the analysis. To ameliorate this risk, the analysis was discussed and cross-checked with other researchers to enhance credibility. The fact that only 10 studies were included in this review increases the risk of bias. Another weakness of this review was the vagueness of the concept of compassionate leadership, which may have influenced the literature search, data collection and analysis.

## Conclusions

This review complements the field of research into a softer-skilled and human-centred approach to health-care leadership research by presenting seven core elements that combine leaders’ and professionals’ experiences and perceptions of compassionate leadership in health care.

The findings show that compassionate leadership has a broad and diverse entirety, and it diverges from other leadership styles by emphasising compassion. Compassionate leadership is comprehensive, and it involves many aspects that need to be taken into consideration in health care. Compassionate leadership was experienced through many aspects, from empathy to enhanced work circumstances. A compassionate leader is there for others and works as an example. Compassionate leadership can also be exhausting, and leaders should know their own limitations. This could be addressed with self-help abilities, as they have a significant impact on becoming compassionate leaders. Contradictories in the findings indicate that compassionate leadership is influenced by situational factors. Health-care organisations face many human resource-related challenges, and organisations could benefit from compassionate leadership, as it has the potential to enhance work environments and work well-being. The findings provide a synthesised knowledge of compassionate leadership and its benefits, which can possibly address challenges like health-care professionals’ burnout, turnover and the lack of patient safety. It should be taken into consideration by health-care leaders, their education and when developing organisational effectiveness.

Future studies should focus on the implementation and evaluation of compassionate leadership in different health-care organisations. There is a need to support and educate leaders for compassionate leadership and systematic development and assessment of the effectiveness of interventions.

## Figures and Tables

**Table 1.** Databases, search strategy and results by databases

Databases	Search strategy	*n*
PubMed	“Leadership”[Mesh] OR “Administrative Personnel”[Mesh] OR leader* OR supervisor* OR manager* OR chief* OR executive* OR director* OR administrator* OR “Administrative Personnel” N2 “Health Occupations”[Mesh] OR “Health Services Administration”[Mesh] OR (nurs* OR health OR healthcare OR hospital OR physician* OR ward*) N3“Empathy”[Mesh] OR empath* OR compassion*	3,288
CINAHL	(MH “Administrative Personnel+”) OR (leader* OR supervisor* OR manager* OR chief* OR executive* OR “head nurse*” OR “charge nurse*” OR administrator* OR director*) N2 (MH “Compassion”) OR (MH “Empathy”) OR empath* OR compassion* N3 (MH “Health Occupations+”) OR (nurs* OR health OR healthcare OR hospital OR physician* OR ward*) OR (MH “Health Services Administration+”)	2,211
Scopus	leader* OR supervisor* OR manager* OR chief* OR executive* OR director* OR administrator* OR “Administrative Personnel” N2 (nurs* OR health OR healthcare OR hospital OR physician* OR ward*) N3 empath* OR compassion*	2,979
Medic	joht* esi* manager* admist* director* osastonh* hallin* N2 compass* empath* myötätun* empat*	43
MedNar	compassionate leader* OR manager*	965

Notes: Searches conducted 4 January 2023, limited to English, Finnish or Swedish. No date limitations applied

**Source:** Authors’ own work

**Table 2.** Inclusion and exclusion criteria using PICo

PICo	Inclusion criteria	Exclusion criteria
Participants (P)	Health-care leaders and professionals	Health-care students
Phenomena of interest (I)	Participants' own experiences of compassionate leadership in qualitative studies or perceptions of pre-defined dimensions of compassionate leadership measured in quantitative studies	No documentation on experiences or perceptions of compassionate leadership
Context (Co)	Health-care organisations in any geographic locations	
Types of studies	Peer-reviewed original articles published in English, Finnish or Swedish No date limit	All types of reviews, nonoriginal articles and all other publications

**Source:** Authors’ own work

**Table 3.** Health-care professionals’ and leaders’ experiences and perceptions of compassionate leadership

Descriptive themes (*n* = 55)	Sub-themes (*n* =18)	Analytical themes (*n* = 7)
Treating professionals as human beings Focusing on the personality of individuals and knowing them A feeling that private needs are met	Leader considers individuality	Treating professionals as individuals with an empathetic and an understanding approach
Showing empathy Having emotional intelligence Being compassionate towards professionals Not receiving empathy or being allowed to show negative feelings	Leader considers empathy	
The feeling that professionals are being understood Being aware of the work and its demands The burden of understanding Missing or inadequate understanding	Leader considers understanding	
Professionals feeling heard Mutual listening	Skills in listening	Building a culture for open and safe communication
Sincere conversations Promotes communication and information sharing Difficulties in communication	Encourages open and authentic discussion	
Being attentive and physically present at the workplace Knowing what is going on in the work unit Working in cooperation with professionals	Presence and participation	Being there for professionals
Being easy to approach Difficulties in approachability Being open to different views Personal virtues of the leader	Being approachable and open	
Supporting the combining of work and personal life Being helpful Giving support to professionals	Personalised support	Giving all-encompassing support
Supporting professional development Building the confidence and resilience of professionals Challenging professionals in a positive way, giving positive and constructive feedback Support is lacking or inadequate	Support in professional growth	
Giving appreciation Giving recognition Making professionals feel valued Making professionals feel empowered Encouraging professionals	Making professional feel meaningful	
Acting as a role model and leading by example Demonstrating compassionate care Impulsive and inconsistent leading	Being a mentor and a good example	Showing the way as a leader and as a strong professional
Solving problems and resolving conflicts Sensitive approach to mistakes	Open and transparent handling of difficult situations	
Standing up for professionals Acting as a professional	Being advocative and a fair professional	
Actively promoting teamwork Recognition and use of team members’ strengths Not recognising employee or team strengths	Knowledge of the team and use of the team in an adequate way	Building circumstances for efficient work and better well-being
Building an environment of respect Building a united work community Improving the quality of care and patient safety	Enhancing the operational environment	
Improving contentment and decreased risk of burnout Improved manager–staff relationships	Relation to overall well-being	
Having experience in leadership Having leadership education and personal development	Building compassionate leadership skills	Growing into a compassionate leader
To know oneself and taking care of oneself The ability to be self-compassionate	Having self-help abilities	

**Source:** Authors’ own work

## Supplementary material

The supplementary material for this article can be found online.
