# Supplementary material for: Health-care leaders’ and professionals’ experiences and perceptions of compassionate leadership: A mixed-methods systematic review
Source: Leadersh Health Serv (Bradf Engl). 2023 Oct 16;37(5):49–65. doi: 10.1108/LHS-06-2023-0043 (PMC10868663; doi:10.1108/LHS-06-2023-0043)
Supplement: Supplementary file 2 [file leadershhealthserv-37-0049-s002.docx]

Supplementary Table II. Excluded studies following full-text review.

| Excluded study | Reason for exclusion |
| --- | --- |
| 1. Benevene, P., Buonomo, I., and West, M. (2022), "Editorial: Compassion and compassionate leadership in the workplace", *Frontiers in Psychology,* Vol. 13, 1074068. | Not primary research |
| 2. Binagwaho, A. (2020), "We Need Compassionate Leadership Management Based on Evidence to Defeat COVID-19", *International Journal of Health Policy and Management*, Vol. 9, No. 10, pp. 413-414. | Not primary research |
| 3. Camilleri, C. (2021), "My role is to provide inclusive and compassionate leadership", *Nursing Times* Vol. 117, No. 12, pp. 51-51. | Not primary research |
| 4. Chen, C., Pao, LS. and Lei, H. (2022), "The examination of job separation tendency  of nursing staff in the first public–private joint-venture hospital in Taiwan: a multiple  mediation model of job satisfaction and job performance", *Humanity Social Sciences Commununity,* Vol 9, No. 425. | Wrong outcomes |
| 5. de Zulueta, P. C. (2015), "Developing compassionate leadership in health care: an integrative review", *Journal of Healthcare Leadership*, Vol. 8, pp. 1-10. | Wrong study design |
| 6. Dougan, C., Philips, S. and Hughes, D. (2021), "Compassionate leadership during COVID-19: an ABC approach to the introduction of new medical graduates as  Foundation interim Year 1s (FiY1s)", *BMJ Leader,* Vol. 2021, No. 5, pp. 199-202. | Wrong outcomes |
| 7. Edwards, L.D., Till A. and McKimm, J. (2018), "Meeting today’s healthcare  leadership challenges: is compassionate, caring and inclusive leadership the answer?",  *BMJ Leader,* Vol 2018, No. 2, pp. 64-67. | Not primary research |
| 8. Evans, D. (2022), "So close to love: compassionate leadership in healthcare", *British Journal of Healthcare Management,* Vol 28, No. 4, pp. 1-8. | Wrong study design |
| 9. Ezzat, A. (2010), "Compassionate leadership", *Journal of Obstetrics and Gynaecology Canada,* Vol. 32, No. 9, pp. 823-825. | Not primary research |
| 10. Foster, S. (2021), "Finding the right leadership style", *British Journal of Nursing,*  Vol. 30, No. 1, pp. 83. | Not primary research |
| 11. Harrel, E., Berland, L., Jacobson, J. and Addiss, D. G. (2021), "Compassionate Leadership: Essential for the Future of Tropical Medicine and Global Health",  *The American Journal of Tropical Medicine and Hygiene*, Vol. 105, No. 6,  pp. 1450-1452. | Not primary research |
| 12. Harrington, A. (2021), "Understanding effective nurse leadership styles during the COVID-19 pandemic", *Nursing Standard,* Vol. 36, No. 5, pp. 45-50. | Not primary research |
| 13. Hornett, M. (2012), "Compassionate leadership", *British Journal of Nursing,* Vol. 21, No.13. pp. 831. | Not primary research |
| 14. Lazarus, A. (2003), "The dead moose and other reflections on compassionate  physician leadership", *Physician Executive*, Vol. 29. No. 3, pp. 37-39. | Not primary research |
| 15. Lewis, C. (2103), "Leadership with Compassion: Applying Kindness, Dignity and Respect in Healthcare Management", *Operating Theatre Journal,* Vol. 12, No. 279,  pp. 12-12. | Not primary research |
| 16. Lown, B. A. (2014), "Toward more compassionate healthcare systems Comment on "Enabling compassionate healthcare: perils, prospects and perspectives". *International Journal of Health Policy and Management*, Vol. *2, No.* 4, pp. 199-200. | Not primary research |
| 17. Lown, B. A. (2018), "Mission Critical: Nursing Leadership Support for Compassion  to Sustain Staff Well-being", *Nursing Administration Quarterly*, Vol. 42, No. 3,  pp. 217-222. | Not primary research |
| 18. McClelland, L. E. and Vogus, T. J. (2021), "Infusing, sustaining, and replenishing compassion in health care organizations through compassion practices", *Health Care Management Review*, Vol. 46, No. 1, pp. 55-65. | Not compassionate leadership |
| 19. McComiskey, F. (2017), "The fundamental managerial challenges in the role of a contemporary district nurse: A discussion", *British Journal of Community Nursing*,  Vol. 22, No. 10, pp. 489-494. | Not primary research |
| 20. McKimm, J., and O'Sullivan, H. (2013), "Personality, self-development the compassionate leader", *British Journal of Hospital Medicine,* Vol. 74, No. 6,  pp. 336-339. | Not primary research |
| 21. Muflahi, O., Morley, K. and Coley, M. (2022), "Inspiring others through  compassionate leadership: -the power of partnerships", *British Journal of Healthcare Assistants,* Vol. 16, No. 4, pp. 172-174. | Not primary research |
| 22. Oruh, E.S., Mordi, C., Dibia, C.H. and Ajonbadi, H.A. (2021), "Exploring  compassionate managerial leadership style in reducing employee stress level during  COVID-19 crisis: the case of Nigeria", *Employee Relations*, Vol. 43 No. 6,  pp. 1362-1381. | Wrong setting |
| 23. Papadopoulos, I., Lazzarino, R., Koulouglioti, C., Aagard, M., Akman, Ö., *et al.*  (2020), "Obstacles to compassion-giving among nursing and midwifery managers: an international study", *International Nursing Review*, Vol. 67, No. 4, pp. 453-465. | Not healthcare professionals or leaders |
| 24. Patton, A. (2021), "What Does Leading with Mindfulness and Compassion  Look Like?", *Oncology Issues*, Vol. 36. No. 5, pp. 52-56. | Not primary research |
| 25. Parsons, T. W. 3rd (2021), "The AOA: A Comprehensive House with Compassionate Leaders. Presidential Address to the AOA, June 15, 2020: AOA Critical Issues",  *The Journal of Bone and Joint Surgery.* Vol. 103, No. 21, e85. | Not primary research |
| 26. Pelley, C. (2021), "We need inclusive and compassionate leadership: The pandemic  has shown that leaders willing to learn from experience can create a more productive environment", *Nursing Management,* Vol.28, No. 1, pp. 13-13. | Not primary research |
| 27. Pitman, S. (2020), "Compassionate leadership", *World of Irish Nursing & Midwifery,* Vol. 28, No. 10, pp. 46-47. | Not primary research |
| 28. Ramdeen, B., Trondillo, J., Kent, R. & Collings, C. (2022), "Does compassionate  nurse leadership exist in practice, or just in theory?", *Nursing Standard,* Vol. 37, No. 10,  pp. 13-13. | Not primary research |
| 29. Straughair, C. (2019), "Cultivating compassion in nursing: A grounded theory study  to explore the perceptions of individuals who have experienced nursing care as patients", *Nurse Education in Practice*, Vol. 35, pp. 98-103. | Not healthcare professionals or leaders |
| 30. Tehan, M. (2007), "The Compassionate Workplace: Leading with the Heart", *Illness, Crisis & Loss, Vol. 15, No.* 3, pp. 205-218. | Not healthcare professionals or leaders |
| 31. Tehan, M. and Robinson, P. (2009), "Leading the Way: Compassion in the  Workplace", *Illness, Crisis & Loss*, Vol. 17, No. 2, pp. 93-111. | Not healthcare professionals or leaders |
| 32. Thomas, N.B. (2017), "Leading with Compassion. A track record of success opens  doors, while fostering a culture of goodwill brings respect", *Healthcare Executive,*  Vol. 32, No. 2, pp. 80-81. | Not primary research |
| 33. Trueland, J. (2019), "Compassionate leadership: does it really give nurses a voice?", *Nursing Standard,* Vol. 34, No. 12, pp.38-41. | Not primary research |
| 34. Trueland, J. (2022), "How to make compassionate leadership in nursing a reality: Empathetic and inclusive leaders enable teams to achieve better outcomes for patients  but can be difficult to find in the hierarchical organisations of the NHS", *Nursing Management,* Vol. 10, No. 29(5), pp. 6-8. | Not primary research |
| 35. Vogel, L. (2019), "Doctors must meet change with courage, compassion: incoming  CMA president", *CMAJ: Canadian Medical Association Journal,*  Vol. 191, No. 35, e972. | Not primary research |
| 36. Vogel, S. and Flint, B. (2021), "Compassionate leadership: how to support your team when fixing the problem seems impossible", *Nursing Management*, Vol. 28, No. 1,  pp. 32-41. | Wrong phenomena |
| 37. Walsh, K. (2020), "Compassionate leadership", *Yoga Journal,* Vol. 1, No. 318,  pp. 72-79. | Not primary research |
| 38. West, M.A. (2019), "Compassionate leadership in health and care settings", In  Galiana, L. and Sansó, N. (Eds.) *The Power of Compassion,* pp*.* 317-338. | Not primary research |
| 39. West, M.A. and Chowla, R. (2017), "Compassionate leadership for compassionate  health care", *Compassion: Concepts, Research and Applications,* pp*.* 237-257. | Not primary research |
| 40. Willis, S. and Anstey, S. (2019), "Compassionate leadership in district nursing:  a case study of a complex wound", *British journal of Community Nursing*, Vol 24, No 2,  p. 50-57. | Not professionals |
| 41. Wollenburg, K. G. (2004), "Leadership with conscience, compassion, and  commitment", *American Journal of Health-system Pharmacy,* Vol. 61. No. 17,  pp.1785-1791. | Not primary research |

**Source**: Authors’ own work
