# Supplementary material for: Health-care leaders’ and professionals’ experiences and perceptions of compassionate leadership: A mixed-methods systematic review
Source: Leadersh Health Serv (Bradf Engl). 2023 Oct 16;37(5):49–65. doi: 10.1108/LHS-06-2023-0043 (PMC10868663; doi:10.1108/LHS-06-2023-0043)
Supplement: Supplementary file 3 [file leadershhealthserv-37-0049-s003.docx]

Supplementary Table III. Quality appraisal for qualitative studies.

| Study | Q1 | Q2 | Q3 | Q4 | Q5 | Q6 | Q7 | Q8 | Q9 | Q10 | Total |
| --- | --- | --- | --- | --- | --- | --- | --- | --- | --- | --- | --- |
| Ali and Terry 2017 | Y | Y | Y | Y | Y | N | N | Y | Y | Y | 8/10 |
| Hewison *et al.,* 2018 | Y | Y | Y | Y | Y | N | N | Y | Y | Y | 8/10 |
| Hewison *et al.,* 2019 | Y | Y | Y | Y | Y | N | N | Y | Y | Y | 8/10 |
| O’Toole *et al.,* 2021 | Y | Y | Y | Y | Y | N | Y | Y | Y | Y | 9/10 |
| Salminen-Tuomaala and Seppälä, 2022b | N | Y | Y | Y | Y | N | U | Y | Y | Y | 7/10 |

*Note:* Q1: Is there congruity between the stated philosophical perspective and the research methodology? Q2: Is there congruity between the research methodology and the research question or objectives? Q3: Is there congruity between the research methodology and the methods used to collect data? Q4: Is there congruity between the research methodology and the representation and analysis of data? Q5: Is there congruity between the research methodology and the interpretation of results? Q6: Is there a statement locating the researcher culturally or theoretically? Q7: Is the influence of the researcher on the research, and vice- versa, addressed? Q8: Are participants, and their voices, adequately represented? Q9: Is the research ethical according to current criteria or, for recent studies, and is there evidence of ethical approval by an appropriate body? Q10: Do the conclusions drawn in the research report flow from the analysis, or interpretation, of the data?

Abbreviations: *N*, No; *U*, Unclear; *Y*, Yes.

**Source**: Authors’ own work
