# Supplementary material for: Health-care leaders’ and professionals’ experiences and perceptions of compassionate leadership: A mixed-methods systematic review
Source: Leadersh Health Serv (Bradf Engl). 2023 Oct 16;37(5):49–65. doi: 10.1108/LHS-06-2023-0043 (PMC10868663; doi:10.1108/LHS-06-2023-0043)
Supplement: Supplementary file 4 [file leadershhealthserv-37-0049-s004.docx]

Supplementary Table IV. Quality appraisal for quantitative studies.

| Study | Q1 | Q2 | Q3 | Q4 | Q5 | Q6 | Q7 | Q8 | Total |
| --- | --- | --- | --- | --- | --- | --- | --- | --- | --- |
| López-Díaz *et al.,* 2022 | Y | Y | Y | Y | N | N | Y | Y | 6/8 |
| Papadopoulos *et al.*, 2021 | Y | Y | Y | Y | N | N | Y | Y | 6/8 |
| Papadopoulos *et al.,* 2022 | Y | Y | Y | Y | N | N | Y | Y | 6/8 |
| Salminen-Tuomaala and  Seppälä, 2022a | Y | Y | Y | Y | N | N | Y | Y | 6/8 |
| Sansó *et al.* 2022 | Y | Y | Y | Y | Y | Y | Y | Y | 8/8 |

*Note:* Q1: Were the criteria for inclusion in the sample clearly defined? Q2: Were the study subjects and the setting described in detail? Q3: Was the exposure measured in a valid and reliable way? Q4: Were objective, standard criteria used for measurement of the condition? Q5: Were confounding factors identified? Q6: Were strategies to deal with confounding factors stated? Q7: Were the outcomes measured in a valid and reliable way? Q8: Was appropriate statistical analysis used?

Abbreviations: *N*, No; *U*, Unclear; *Y*, Yes.

**Source**: Authors’ own work
