# Supplementary material for: Health-care leaders’ and professionals’ experiences and perceptions of compassionate leadership: A mixed-methods systematic review
Source: Leadersh Health Serv (Bradf Engl). 2023 Oct 16;37(5):49–65. doi: 10.1108/LHS-06-2023-0043 (PMC10868663; doi:10.1108/LHS-06-2023-0043)
Supplement: Supplementary file 5 [file leadershhealthserv-37-0049-s005.docx]

Supplementary Table V. Data extraction table for included qualitative and quantitative studies.

| Author(s), year, and country | Purpose | Participants and context | Methodology (design, data collection, data  analysis) | Key findings | Quality  appraisal |
| --- | --- | --- | --- | --- | --- |
| Ali and Terry, 2017,  United Kingdom | To understand how leaders and senior staff within a Community NHS Trust perceive compassionate leadership and its importance. | Nurse leaders (n = 11) from Community NHS Trust who had compassionate leadership as a  key aspect of their job role. | A qualitative study design. Semi-structured face-to-face interviews.  Interpretative phenomenological analysis (IPA). | Compassionate leadership involves role modelling, value-driven person-centered care and compassion towards both staff and patients. Leading by example was considered important as it can shape staff behavior.  Compassionate leadership was described as leading with both the head and the heart.  Compassionate leadership can be used to ensure that patients, clients, and families feel cared for, not merely treated. | JBI Critical Appraisal Checklist for Qualitative Research 8/10 |
| Hewison *et al.,* 2018,  United Kingdom | To report an evaluation of a leading-with-compassion recognition  scheme and to present a new framework for compassion derived from the data. | Staff members (n = 8) from the ten participating organizations with knowledge of the design and  implementation of the scheme, and who had all nominated someone for recognition were interviewed. Participating organizations were situated in Shropshire and Staffordshire, United Kingdom. | A retrospective review.  Qualitative semi-structured interviews, a focus group, and thematic data  analysis. Content analysis of 1,500 nominations of compassionate acts. | Compassion towards staff and patients is important.    Compassionate leadership is described as role modelling, supporting, listening and as appreciation of staff. Compassionate leadership leads to direct improvement in patient care. | JBI Critical Appraisal Checklist for Qualitative Research 8/10 |
| Hewison *et al.,* 2019,  United Kingdom | To explore compassionate leadership with those involved in leading system-wide end-of-life care. To define compassionate leadership in the context of  palliative and end-of-life care; collect accounts of compassionate leadership activity from key stakeholders in end-of-life and palliative care and identify examples of compassionate leadership in practice. | Staff (n = 14) from healthcare  organizations including hospitals, hospices and community teams. The participants were working in palliative and end-of-life care (PEolC) units. | A qualitative study design. Focus group interviews.  Thematical analysis. | It is important that the leadership role is both role modelling and nurturing.  Challenging others was described as a crucial element of leadership.  Leaders must manage boundaries effectively.  Supporting staff is considered important. | JBI Critical Appraisal Checklist for Qualitative Research 8/10 |
| López-Díaz *et al.,* 2020,  Colombia | To recognize compassionate practices and the obstacles  to their use by nurse managers in Colombia. | Nurse managers (n = 69) from Colombia. | Mixed cross-sectional, descriptive, exploratory study.  Quantitative data  analyzed with descriptive statistics, qualitative data processed using the Nvivo software and thematic analysis.  Part of a bigger international study. | Compassionate leaders listen, support, and recognize staff individually.  Compassionate leadership creates a supportive and harmonious work environment.  Most of the nurse managers received compassion and they could understand its importance. | JBI Critical Appraisal Checklist for Analytical Cross-sectional Studies 6/8 |
| O’Toole *et al.,* 2021,  Australia | To examine the perceptions of leadership by leaders within residential aged  care and to identify the crucial requirements for successful leadership in this complex industry. | Senior managers (n = 18) working in residential aged care. | A qualitative study design.  Semi-structured interviews.  Thematic analysis. | Diversity of leadership skills is required. Communication and compassion are important in leadership.  Senior staff in the aged care industry believe that compassion is both a key component and a gap in the skill set of aged care leadership and leaders.  Compassionate caring is central to high-quality care in residential aged care. | JBI Critical Appraisal Checklist for Qualitative Research 9/10 |
| Papadopoulos *et al.,* 2021,  United Kingdom/ international study | To explore the views of nursing and midwifery managers from  different countries in relation to the definition, advantages, and importance of compassion. | Nursing  and midwifery managers (n = 1,217 across 17 countries) | A cross-sectional,  descriptive, exploratory online survey.  Hybrid approach of inductive and deductive thematic analysis. | Compassion towards managers was described as feeling valued and respected. Giving compassion turned out receiving understanding, support, and appreciation. Importance of being a role model for their staff. Compassion was considered an important part in their leadership style for some.  The benefits of compassionate leadership were considered as more positive, and open staff relations. Compassion towards staff also enhanced teamwork, improved communication and participatory, problem-solving, decision-making and conflict resolution. Compassion was overall regarded beneficial for both managers and staff’s mental health, improving their professional life quality and satisfaction, while decreasing the risk of burnout. | JBI Critical Appraisal Checklist for Analytical Cross-sectional Studies 6/8 |
| Papadopoulos *et al.,* 2022,  United Kingdom/ international study | To explore the views of an international sample of  nursing and midwifery managers concerning attributes that they associate with compassionate management. | Nursing  and midwifery managers (n = 1,217 across 17 countries) | A cross-sectional online survey.  Open-ended questions.  Content analysis.  A relative distribution  of the identified themes for the overall sample and for each  participating country was calculated. | Compassionate leaders were described with six main attributes. Virtuous support included awareness, active support towards staff members. Communication included an available and an approachable, sensitive, active listener.  Personal virtues included empathy, understanding, accepting and a non-judging attitude. It also described a compassionate leader as a warm leader who is considerate, attentive, kind, and amiable.  A compassionate leader was also described as a participating leader who supports personal development and teamwork. | JBI Critical Appraisal Checklist for Analytical Cross-sectional Studies 6/8 |
| Salminen-Tuomaala and Seppälä, 2022a,  Finland | To investigate how intensive and emergency nurses  rated the adequacy of compassionate leadership during the early stages of the  Covid-19 pandemic. | Intensive and emergency care nurses (n = 50) in a central hospital in Finland. | A quantitative descriptive cross-sectional study.    Statistic data analyzation. | There was a greater need for compassionate leadership during the Covid-19, but it was not received by nurses.  Provided support was unequal while understanding and encouraging were not provided. Competence was not appreciated, and personal development not supported.  Empathy was considered important, but it was not visible.    Compassionate leadership can be developed through experience, personal development, and education. | JBI Critical Appraisal Checklist for Analytical Cross-sectional Studies 6/8 |
| Salminen-Tuomaala and Seppälä, 2022b,  Finland | To provide a trustworthy description of nurses' experiences and expectations for  compassionate leadership and compassion at a central hospital in Finland. | Intensive and emergency care nurses (n = 50) in a central hospital in Finland. | An online survey tool with open questions.  Inductive content analysis. | There were many positive experiences of compassion including empathy, participation, listening and understanding. There was also an absence of compassionate leadership which was described as missing support, not being understood and as difficulties in communication.    The nurses’ expectations included individual attention and genuine physical and psychological presence from their leaders.    The benefits of compassionate leadership include enhanced wellbeing, reduced stress, improved psychological safety and improved patient safety. | JBI Critical Appraisal Checklist for Qualitative Research 7/10 |
| Sansó *et al.,* 2022,  Spain | To adapt and validate the Compassionate  Leadership Self-reported Scale in a sample of palliative care professionals; and to  study the relation between compassionate leadership and associated concepts of self-compassion, awareness, and self-care. | Spanish healthcare professionals working in end-of-life care (n = 296). | A cross-sectional survey.  Descriptive statistics analysis. Confirmatory factor analysis (CFA) with four-correlated factors, reliability estimates and a structural model. | Compassionate leadership is regarded as attending, understanding, empathizing, and helping.  The structural model predicting compassionate leadership suggested that the dimensions of attending and understanding were most highly related to positive self-compassion and awareness.  Empathizing was related to self-care and awareness while helping was related to positive self-compassion and self-care. | JBI Critical Appraisal Checklist for Analytical Cross-sectional Studies 8/8 |

**Source**: Authors’ own work
