# Supplementary material for: Health-care leaders’ and professionals’ experiences and perceptions of compassionate leadership: A mixed-methods systematic review
Source: Leadersh Health Serv (Bradf Engl). 2023 Oct 16;37(5):49–65. doi: 10.1108/LHS-06-2023-0043 (PMC10868663; doi:10.1108/LHS-06-2023-0043)
Supplement: Supplementary file 6 [file leadershhealthserv-37-0049-s006.docx]

Supplementary Table VI. The qualitizing of quantitative results.

| Study | Textual description | Code |
| --- | --- | --- |
| Salminen-Tuomaala and Seppälä 2022a | 22% fully agreed and 44% somewhat agreed that the  Covid-19 pandemic increased the need for compassion in their work community | Stressful times increase the need for compassion in the work community |
|  | 20% fully disagreed and 36% somewhat disagreed that they  had received support from their immediate supervisor during  the Covid-19 pandemic | Received support during stressful times is inadequate |
|  | 22% fully disagreed and 38% somewhat disagreed that the supervisor of the work community supports everybody equally | Support from the leader is unequal |
|  | 26% fully disagree and 34% somewhat disagree that the supervisor encourages employees in acute situations | Encouragement in acute situations is inadequate |
|  | 26% fully disagree and 34% somewhat disagree that the supervisor supports employees in acute and challenging situations | Support in acute and challenging situations is inadequate |
|  | 28% fully disagree and 36 % somewhat disagree that the supervisor can encourage employees in acute situations | Encouragement in acute situations is inadequate |
|  | 20% fully disagree and 38% somewhat disagree that the supervisor understands employees’ insecurity in acute  situations | The leader does not understand employees’ insecurity in acute situations |
|  | 76% fully agree and 18% somewhat agree that empathy is important in working life | Empathy is important in working life |
|  | 24% fully disagree and 44% somewhat disagree that empathy  is visible in the work of the supervisor | Empathy is not visible enough in leaders’ work |
|  | 29% fully disagree and 32% somewhat disagree that the supervisor of the work community allows the expression of negative emotions | Negative emotions are not allowed |
|  | 14% fully disagree and 36% somewhat disagree that their immediate supervisor is easy to approach. 16% fully agree and 28% somewhat agree that their immediate supervisor is easy to approach | Approachability is not always easy |
|  | 16% fully disagree and 30 % somewhat disagree that everybody’s competence is appreciated in their work  community. 30% could not say | Everybody’s competence is not appreciated |
|  | 16% fully disagree and 30 % somewhat disagree that the supervisor appreciates everybody’s competence in their work community. 30% could not say | The leader does not appreciate everybody’s competence |
|  | 16% fully disagree and 40% somewhat disagree that  everybody’s development is equally supported in the work community | Everybody’s development is not equally supported |
|  | 12% fully disagree and 40% somewhat disagree that supervisor recognizes the strengths of the work community members | The leader does not recognize the strengths of the work community members |
|  | 16% fully disagree and 36% somewhat disagree that the supervisor makes use of work community members’ strengths  to reach the common objective | The leader does not make use of work community members’ strengths to reach the common objective |
|  | 12% fully agree and 54% somewhat agree that the team recognizes members’ strengths required to reach the common objective | The team recognizes members’ strengths required to reach the common objective |
|  | 14% fully agree and 52% somewhat agree that the team makes use of members’ strengths to reach the common objective | The team makes use of members’ strengths to reach the common objective |
|  | 26% fully agree and 54% somewhat agree that compassionate leadership is reached through experience | Compassionate leadership can be reached through experience |
|  | 32% fully agree and 48% somewhat agree that compassionate leadership is reached through personal development | Compassionate leadership can be reached through personal development |
|  | 44% fully agree and 12% somewhat agree that compassionate leadership can be developed through education | Compassionate leadership can be developed through education |
| Sansó et al. 2022 | Items were scored in a 5-point Likert scale, ranging from 1 (totally disagree) to 5 (totally agree) |  |
|  | Listen carefully when exploring problems (mean 4.21) | Careful listening when solving problems |
|  | Pay close attention when listening (mean 4.18) | Attentive listening |
|  | Are very attentive when a member of the team tell them  about difficulties (mean 4.33) | Attentive listening to difficulties |
|  | Give full attention when members of the team describe challenges they face (mean 4.30) | Being attentive |
|  | Are helpful in understanding the causes of difficulties the  team faces (mean 4.17) | Understanding approach |
|  | Do not impose their understanding of the causes of difficulties the team faces (mean 3.80) | Not blaming anything or anyone |
|  | Take time to understand carefully the causes of the problems (mean 4.02) | Trying to understand problems |
|  | Work together with the team to come to an understanding of problems (mean 4.11) | Working together with the team |
|  | Are genuinely warm and empathic (mean 4.05) | Being warm  Being empathetic |
|  | Are emotionally in touch with others’ feelings when they are upset (mean 3.99) | Putting yourself in others’ shoes |
|  | Are sensitive to what others are feeling (mean 4.07) | Emotional intelligence |
|  | Genuinely care about others’ difficulties (mean 4.13) | Compassionate approach |
|  | Help people practically with problems they face (mean 4.02) | Helping others with their problems |
|  | Take effective action to help others with the problems they  face (3.91) | Taking effective actions to help |
|  | Deal effectively with problems in order to help others (mean 3.89) | Solving problems to help others |
|  | Are genuinely committed to making a difference by serving others (mean 4.00) | Making a difference by serving others |
|  | Positive self-compassion would refer to compassionate self-responding to personal struggle (mean 3.40) | Having positive self-compassion |
|  | Negative self-compassion would refer to uncompassionate  self-responding to personal struggle (berating oneself for  feeling bad or repressing negative feelings) (mean 2.80) | Not having negative self-compassion |
|  | The general tendency to be aware and conscious of one's own experiences of daily life (mean 4.00) | Having self-awareness |
|  | Self-care is composed by nine items and assesses three dimensions of professionals’ self-care: physical, which refers  to the implication in activities that helps to maintain a healthy body; inner, which is related to activities that help to keep a healthy mind; and social, regarded to activities related to  social activities that help the individual to maintain social  health (mean 3.63) | Taking actions for psychical and psychological self-care |

**Source**: Authors’ own work
